# Oligochitosan-stabilized photoluminescent gold nanoconstructs for optical bioimaging

**DOI:** 10.1186/s40824-017-0107-5

**Published:** 2017-10-17

**Authors:** Donghyuck Yoo, Dongwon Lee

**Affiliations:** Department of BIN Convergence Technology, Chonbuk National University, Chonbuk, Jeonju, 567-756 South Korea

**Keywords:** Gold nanoconstruct, Oligochitosan, Nanocomplexes, Fluorescence imaging

## Abstract

**Background:**

Gold nanoclusters (AuNCs) are typically composed of several to tens of gold atoms which are stabilized with biomacromolecules such as bovine serum albumin (BSA). Au NCs fluoresces in the visible to near infrared region, in a size-dependent manner. AuNCs solutions have potential as fluorophore in a wide range of biomedical applications such as biodetection, biosensing and bioimaging in vitro and in vivo*.* However, their stability and harsh condition of preparation limit their biomedical application.

**Methods:**

BSA stabilized AuNCs (BSA-AuNCs) were prepared by mixing HAuCl_4_ solution with BSA solution for 24 h at 37°C under basic condition. BSA-AuNCs were then mixed with oliogochitosan (OCS) to generate BSA-Au-OCS nanocomplexes. The physicochemical and optical properties of BSA-Au-OCS nanocomplexes were studied using a fluorospectrometer. Their potential as a bioimaging agent in vivo and in vitro was evaluated using a fluorescent imaging instrument.

**Results:**

BSA-stabilized AuNCs solutions were mixed with oligochitosan (OCS) to develop BSA-Au-OCS nanocomplexes of a mean diameter of ~250 nm. BSA-Au-OCS nanocomplexes could emit light at 620 nm and the complexation with OCS did not affect the photophysical properties of BSA-AuNCs. BSA-Au-OCS nanocomplexes showed less cytotoxicity than BSA-AuNCs and was readily taken up by cells. BSA-Au-OCS nanocomplexes showed strong fluorescence in tissues.

**Conclusions:**

We developed stable BSA-Au-OCS nanocomplexes which fluoresce in the near infrared region. BSA-Au-OCS nanocomplexes exhibited significantly less cytotoxicity and strong fluorescence emission, suggesting potential for biomedical applications.

## Background

In recent years, noble metal nanoconstructs have been extensively employed in biomedical applications such as diagnosis and therapeutics due to their unique properties of small size, large surface area to volume ratio, and excellent stability [1–4]. Noble metal nanoconstructs exhibit unique optical properties which render them highly and extensively useful for imaging applications [5–7]. Among numerous noble metals, gold is one of the most commonly studied because of its stable chemical property, biocompatibility and non-immunogenicity [3]. In particular, gold has been used in the treatment of rheumatoid arthritis [8–10]. Gold is easily formulated in various shapes and different sizes such as nanoparticles, nanorods, nanowires, nanocages and nanoclusters [11]. These fascinating aspects made gold nanoconstructs one of key materials of nanoscience and nanotechnology [3, 4].

Gold nanoclusters (AuNCs) are typically composed of several to tens of gold atoms and have a mean diameter of less than ~ 2 nm [12]. AuNCs have emerged as fascinating fluorophore and drawn tremendous attention in biomedical research. Unlike spherical gold nanoparticles which exhibit surface plasmon resonance absorption in the visible region, AuNCs display molecule-like properties and fluoresce in the visible to near infrared region, in a size-dependent manner [1, 13]. The emission wavelength of AuNCs is known to be dependent on the number of atoms in the cluster [7]. In addition, AuNCs have long life-time fluorescence, large two-photon excitation, high emission rate, and large Stokes shift. The mechanism of photoluminescence and photophysical properties of AuNCs has not been clearly understood. However, these unique optical properties establish AuNCs to be a novel fluorophore in a wide range of biomedical applications such as biodetection, biosensing and bioimaging in vitro and in vivo [1, 14, 15].

Over the past decade, several methods have been developed to develop AuNCs [2, 4, 16, 17]. The critical parameter for the synthesis of stable AuNCs and control of photophysical properties is the selection of capping agents and reducing agents, such as thiol compounds, peptides, proteins and polymers [7, 17]. AuNCs prepared using 2-phenylthanethiol exhibited low quantum yield, poor dispersibility and chemical instability [1]. AuNCs synthesized via chemical reduction using sodium borohydride (NaBH_4_) in the presence of glutathione could fluoresce in the blue to near infrared regimes, but with low quantum yield [16, 18]. Poly(amidoamine) dendrimer has been also used as a template to develop AuNCs with high (>10%) quantum efficiency, but the synthesis requires a long time [2, 6]. Recently, a “green” synthetic method has been developed to synthesize stable and dispersible AuNCs using biomacromolecules such as bovine serum albumin (BSA), which acts as a structure-defined scaffold to induce nucleation and growth of AuNCs [2, 12]. BSA is known to coordinate Au^3+^ ions and also convert into Au^3+^ into Au^+^ ions, with the help of tyrosine, aspartate, glutamate, asparagine, and glutamine [1]. BSA is the most commonly used protein for the synthesis of AuNCs as a capping and a reducing agent. Their size and fluorescence emission can be manipulated by varying the molar ratio of protein/Au^3+^, ionic strength, and pH. In a typical synthesis, BSA-stabilized Au nanoclusters (BSA-AuNCs) are prepared at a pH value > 11 to establish strong reducing strength of tyrosine residues [1]. Dry BSA-AuNCs nanocomplexes powders are obtained from freeze drying. However, BSA-AuNCs is dispersed only under basic conditions, which would limit their applications for bioimaging.

**Fig. 1:**
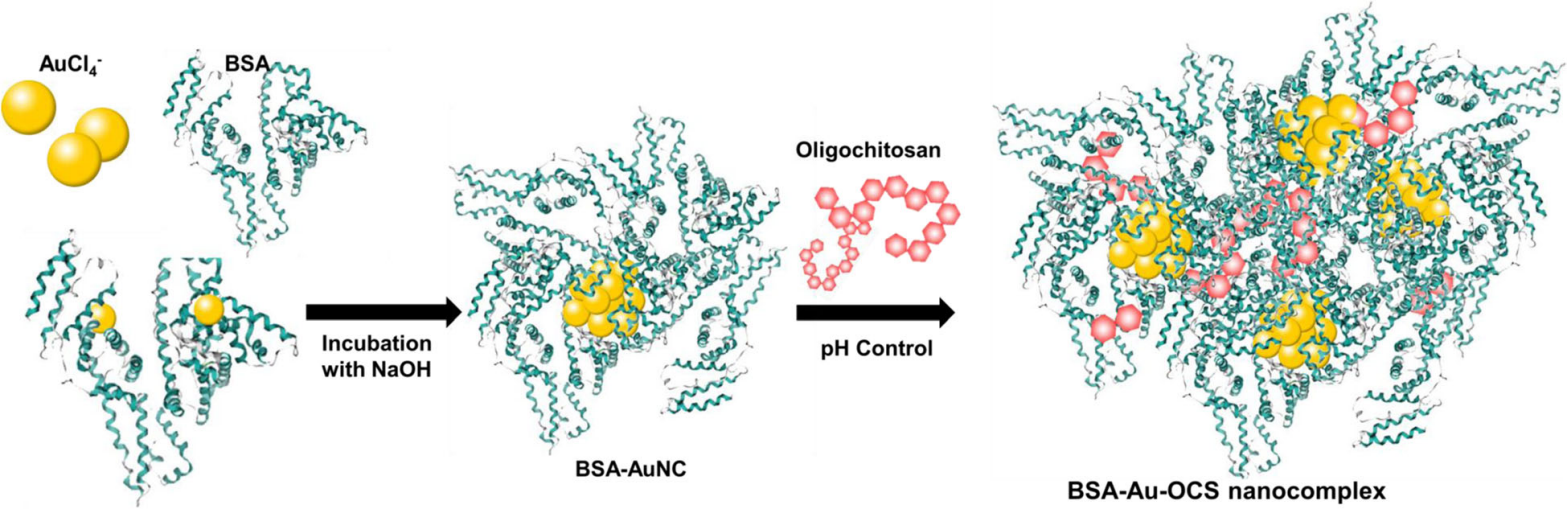
Schematic illustration of BSA-Au-OCS nanocomplexes

The critical issues in biomedical imaging are stability during circulation, affinity toward cells, cellular uptake and toxicity. Despite great advance in the synthesis of AuNCs, the interactions of cells with AuNCs have yet not been understood clearly. In addition, the findings of gold nanoparticles smaller than 50 nm in the studies of cellular uptake and toxicity are limited as the size of AuNCs decreases below 2 nm [12]. Great efforts have also recently been dedicated to modify the BSA-AuNCs with recognition molecules to enhance their performance in bioimaging. In this study, AuNCs were modified with oligochitosan (OCS) to enhance cellular uptake and reduce their toxicity (Fig. 1). Negatively charged BSA-AuNCs formed nanocomplexes with positively charged OCS through electrostatic interactions to generate BSA-Au-OCS nanocomplexes. Herein, we report the optical and physicochemical properties of BSA-Au-OCS nanocomplexes and their potential for bioimaging in vivo and in vitro.

## Methods

### Materials

HAuCl_4_ and BSA were obtained from Sigma-Aldrich (St. Louis, MO, USA). Oligochitosan (Mn: ~1150 Da) was obtained from E-ZE Corp. (Korea). All chemicals were used as received.

### Preparation of BSA-AuNCs

BSA-AuNCs were prepared as previously reported [2]. In brief. HAuCl_4_ solution (5 mL, 5 mM) was added to 5 mL of BSA solution (50 mg/mL) and the mixture was stirred vigorous for 2 min. NaOH (0.5 mL, 1 M) was added to the mixture and the reaction was allowed to generate BSA-AuNCs under stirring for 24 h at 37°C.

### Preparation of BSA-au-OCS nanocomplexes

Water soluble OCS solution (5 mL, 1 mg/mL) was added to 5 mL of BSA-AuNCs solution under vigorous mechanical stirring. Acetic acid solution (900 μL, 1%) was added dropwise to the mixture to reduce pH to 6.0 and the reaction was allowed at room temperature for 6 h. The prepared BSA-Au-OCS nanocomplexes were obtained by centrifugation at 9000×*g* for 10 min and dispersed with 1 mL of distilled water. Solid BSA-Au-OCS nanocomplexes were obtained by freeze drying and stored at 4 °C before use.

### Physicochemical and optical properties of BSA-au-OCS nanocomplexes

The morphology and size of BSA-Au-OCS nanocomplexes were observed by dynamic light scattering (DLS) using a particle size analyzer (90Plus,Brookhaven Instrument Corp., USA) and transmission electron microscope (TEM H7650, HITACHI, Japan). The zeta potential of BSA-Au-OCS nanocomplexes dispersed in distilled water was determined using a particle analyzer (ELS-6000, Ostka, Japan). Fluorescence of BSA-Au-OCS nanocomplexes was studied using a fluorospectrometer (FP-6300, JASCO, Japan).

### Cell culture studies

Cytotoxicity of BSA-AuNCs and BSA-Au-OCS was evaluated by a standard MTT (3-(4,5-Dimethylthiazol-2-yl)-2,5-Diphenyltetrazolium Bromide) assay. NIH3T3 cells (1×10^5^) were cultured in DMEM (Dulbeco’s Modified Eagle’s Medium) for 24 h before treatment. Cells were treated with various concentrations of BSA-AuNCs and BSA-Au-OCS nanocomplexes for 24 h. MTT solution was added to cells and incubated for 4 h. The resulting formazan crystals were dissolved by 1 mL of dimethyl sulfoxide and the absorbance was measured at 570 nm using a microplate reader (Bioteck Instrument, USA).

### Fluorescence imaging of BSA-au-OCS nanocomplexes in vitro and in vivo

Cellular uptake of BSA-Au-OCS was observed using RAW264.7 cells. Cells (1×10^5^) cultured in DMEM were treated with 100 μL of BSA-Au-OCS nanocomplexes (2 mg/mL) for 3 h. Cells were observed under the confocal laser scanning microscope (LSM 510 META, Carl Zeiss, Germany). Fluorescence imaging of BSA-Au-OCS nanocomplexes was obtained using an imaging instrument (IVIS-Spectrum, Caliper Life Science, USA) with excitation at 535 nm and emission at 640 nm. For in vivo fluorescence imaging, 50 μL of BSA-AuNCs or BSA-Au-OCS nanocomplexes (2 mg/mL) were intramuscularly injected into the thigh of mice (hairless SPF/SPF, 8 week, Orient Bio, Korea). Fluorescence imaging was made at 1 min after injection.

## Results

### Synthesis and morphological properties of BSA-au-OCS nanocomplexes

**Fig. 2:**
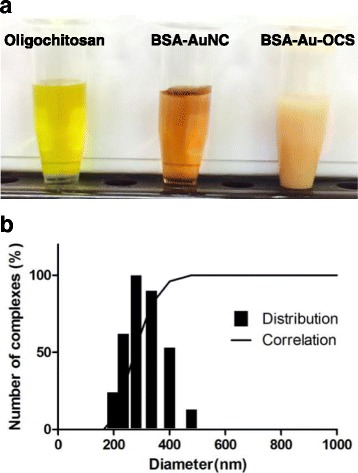
Characterization of BSA-Au-OCS nanocomplexes. **a** Photographs of BSA-Au NCs and BSA-Au-OCS nanocomplexes. **b** Size and size distribution of BSA-Au-OCS nanocomplexes

**Fig. 3:**
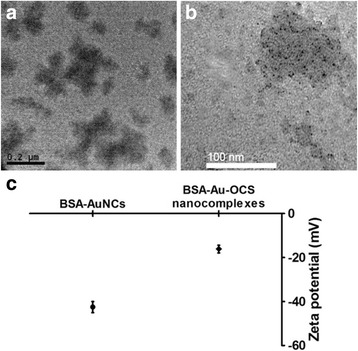
Physicochemical properties of BSA-Au-OCS nanocomplexes. Representative TEM micrographs of BSA-Au NCs (**a**) and BSA-Au-OCS nanocomplexes (**b**). **c** Zeta Zeta potential of BSA-Au NCs and BSA-Au-OCS nanocomplexes. Values are mean±SD (*n* = 4)

NaOH was added to the mixture of BSA and HAuCl_4_ to maximize the reducing capacity of tyrosine residues. Under basic conditions, BSA mediated the formation of AuNCs because histidine residues coordinate with Au^3+^ ions and tyrosine residues efficiently reduces Au^3+^ ions to form AuNCs. During the incubation at 37°C, the color of solution changed from light yellow to brown, indicating the formation of stable AuNCs [11]. At 12 h, the solution exhibited dark brown color. BSA-AuNCs solution was then mixed with OCS at a weight ratio of 25:1 (BSA:OCS) to generate BSA-Au-OCS nanocomplexes which are bright yellow (Fig. 2a). OCS electrostatically interacted with BSA which has an isoelectric point of 4.7 at 25°C and stabilizes AuNCs. BSA-Au-OCS nanocomplexes were freeze-dried and resuspended in deionized water for physicochemical characterization. The pH of BSA-Au-OCS nanocomplexes was ~ 6.8. The average diameter of BSA-Au-OCS nanocomplexes was determined to be ~ 250 nm by DLS (Fig. 2b). TEM image illustrates that AuNCs with an average diameter of 1–5 nm were well distributed and stabilized by BSA (Fig. 3a,b). Before complexation with OCS, BSA-AuNCs solution had Zeta potential of −39 mV. The addition of oppositely charged OCS significantly increased the Zeta potential to −17 mV (Fig. 3c). The results indicate that BSA interacted with OCS electrostatically and the addition of OCS exerts no effects on the formation of AuNCs. After 3 days of incubation under physiological conditions, the BSA-Au-OCS nanocomplexes displayed no change in the hydrodynamic diameter, suggesting that OCS formed highly stable nanocomplexes with BSA-AuNCs.

### Photophysical properties of BSA-au-OCS nanocomplexes

**Fig. 4:**
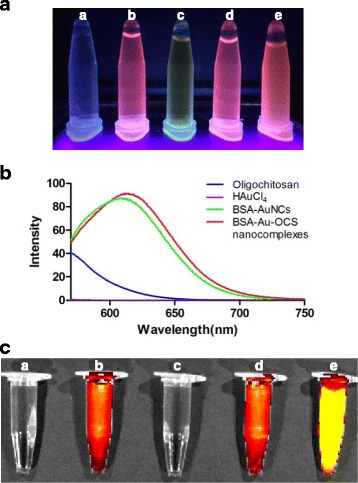
Photophysical properties of BSA-Au-OCS nanocomplexes. **a** Representative photographs of BSA-Au NCs and BSA-Au-OCS nanocomplexes under UV (365 nm) light. a: water, b: BSA-Au NCs, c: oligochitosan solution, d: BSA-Au-OCS nanocomplexes, e: concentrated BSA-Au-OCS nanocomplexes (10×). **b** Fluorescence spectra of BSA-Au NCs and BSA-Au-OCS nanocomplexes at excitation wavelength of 535 nm. **c** Fluorescence imaging of BSA-Au NCs and BSA-Au-OCS nanocomplexes at excitation wavelength of 535 nm and emission wavelength of 640 nm. a: water, b: BSA-Au NCs, c: oligochitosan solution, d: BSA-Au-OCS nanocomplexes, e: concentrated BSA-Au-OCS nanocomplexes (10×)

BSA-AuNCs and BSA-Au-OCS nanocomplexes emitted intense red light under UV (365 nm) light (Fig. 4a). The photophysical property of BSA-AuNCs and BSA-Au-OCS nanocomplexes was studied using a fluorospectrometer. Fig. 4b shows the fluorescence emission of BSA-AuNCs solution and BSA-Au-OCS nanocomplexes at the same concentration of AuNCs. Both BSA-AuNCs and BSA-Au-OCS nanocomplexes exhibited a strong red fluorescence emission at 620 nm, with an excitation wavelength of 535 nm, while negligible fluorescence emission was observed with a BSA solution. Complexation with OCS displayed no effects on the fluorescence intensity of AuNCs, but induced slight red-shift due probably to interaction of fluorophores to OCS. We next investigated the potential of BSA-Au-OCS nanocomplexes as a fluorescent imaging agent using an IVIS imaging instrument. Fig. 4c shows the fluorescence imaging of BSA-Au-OCS nanocomplexes with 535 nm excitation and 640 nm emission. BSA-AuNCs solution and BSA-Au-OCS nanocomplexes exhibited almost the same fluorescence intensity at the same concentration of AuNCs, which is in good agreement with the fluorescence emission (Fig. 4b). Highly concentrated BSA-Au-OCS nanocomplexes showed remarkably strong fluorescence intensity, indicating the concentration-dependent fluorescence.

### Cytotoxicity of BSA-au-OCS nanocomplexes

**Fig. 5:**
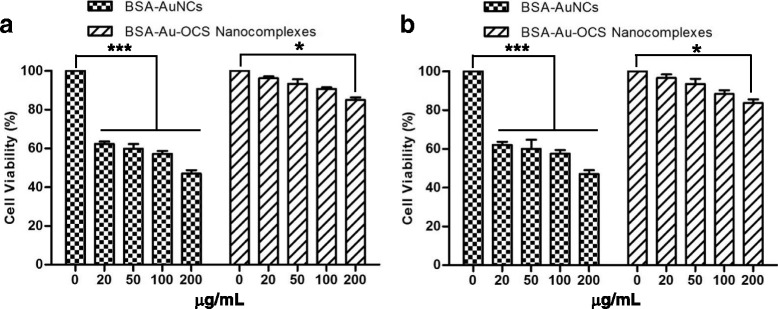
Cytotoxicity of BSA-Au NCs and BSA-Au-OCS nanocomplexes against **a** NIH3T3 and **b** RAW264.7 cells. Values are mean±SD (*n* = 3). *P < 0.05, ***P < 0.001

Cytotoxicity is one of major issues in the development of therapeutic and bioimaging agents [19]. The cytotoxicity of BSA-Au-OCS nanocomplexes was evaluated by standard MTT assay using NIH3T3 and RAW264.7 cells (Fig. 5). BSA-AuNCs solution displayed cytotoxicity at concentrations higher than 20 μg/mL, due probably to the high pH (> 12.0) resulting from the use of NaOH. However, BSA-Au-OCS nanocomplexes showed significantly less cytotoxicity than BSA-AuNCs. The reduced cytotoxicity can be explained by the neutralization and subsequent pH reduction (~ 6.8) by the addition of acetic acid.

### Fluorescence imaging of BSA-au-OCS nanocomplexes in vitro and in vivo

**Fig. 6:**
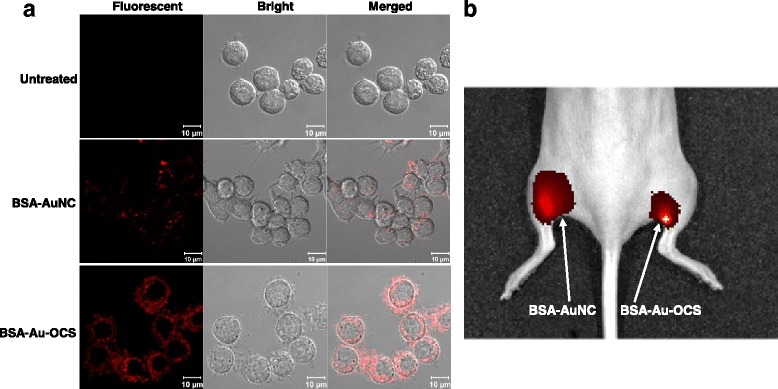
Fluorescence imaging of BSA-Au-OCS nanocomplexes. **a** Confocal laser scanning microscopy of cells treated with BSA-Au NCs or BSA-Au-OCS nanocomplexes. **b** In vivo fluorescence imaging of BSA-Au NCs or BSA-Au-OCS nanocomplexes directly injected into the muscle of mice. Mice were injected with 100 μg of BSA-Au NCs or BSA-Au-OCS nanocomplexes and fluorescence imaging was made with excitation wavelength of 535 nm and emission wavelength of 640 nm

RAW264.7 cells were treated with BSA-AuNCs solution or BSA-Au-OCS nanocomplexes and observed under a confocal laser scanning microscope. As shown in Fig. 6a, marginal fluorescence was observed with cells treated with BSA-AuNCs solution, indicating that BSA-AuNCs was not effectively taken up by cells. However, after incubation with BSA-Au-OCS nanocomplexes, cells showed remarkable red fluorescence in the cytosol, suggesting that oligochitosan significantly decreases negative charge and facilitates internalization of BSA-Au-OCS nanocomplexes.

For in vivo fluorescence imaging, BSA-AuNCs or BSA-Au-OCS nanocomplexes were directly injected into the muscle of a mouse. Fluorescence imaging was obtained with excitation wavelength of 535 nm and emission wavelength of 640 nm, for 10 s of acquisition time. Both BSA-AuNCs and BSA-Au-OCS nanocomplexes displayed strong fluorescence in tissues, demonstrating their potential for bioimaging.

## Discussion

Metal nanoclusters, in particular AuNCs hold great potential as an optical imaging agent in a wide range of biomedical applications and there have been great advances in the development of AuNCs. However, the cellular interactions with AuNCs have yet not been completely elucidated. BSA has been most widely used as a reducing and stabilizing agent for the synthesis AuNCs and is known to accumulate in cells via clathrin-mediated endocytosis and/ or micropinocytosis [20]. However, many authors reported that cellular uptake of BSA-AuNCs is very low and their uptake efficiency can be enhanced by the conjugation of targeting ligands [21, 22]. As shown in Fig. 6, we also found that BSA-AuNCs were not readily taken up by cells and complexation with OCS significantly improved their cellular uptake. Cells showed homogeneously distributed strong fluorescence not only in the plasma membrane but also in the cytoplasm, demonstrating the enhanced cellular uptake.

In this study, water soluble OCS was employed to form nanocomplexes with BSA-AuNCs. OCS was selected because of its excellent biocompatibility, well-documented toxicity profile and polycationic nature. The amino group of chitosan has a pKa value of ~ 6.5 [23] and therefore OCS could form complexes with negatively charged BSA under acidic conditions through electrostatic interactions. Unlike water soluble BSA-AuNCs solutions, BSA-Au-OCS nanocomplexes are solid nanoconstructs in aqueous solutions with a mean diameter of ~ 250 nm, as evidenced by dynamic light scattering (Fig. 2b). The difference in physical status could also explain their different cellular uptake efficiency. We reason that solid BSA-Au-OCS nanocomplexes could be effectively taken up through by non-receptor mediated endocytosis.

We developed highly stable photoluminescent BSA-Au-OCS nanocomplexes by simple addition of water soluble OCS in acetic acid. OCS is reported to be highly soluble even in neutral water and more biocompatible and biodegradable [24]. Before the addition of acetic acid, OCS was completely soluble in highly basic solution of BSA-AuNCs. Addition of acetic acid reduced the pH to 6.8 and OCS formed solid nanocomplexes with BSA-AuNCs. The formation of nanocomplexes was easily observed by the transition from transparent solution to cloudy suspension (Fig. 2a). Cellular uptake, biocompatibility and stability of BSA-AuNCs were significantly enhanced without the deterioration of photophysical properties. However, more mechanistic studies are warranted to elucidate the cellular uptake and toxicity.

## Conclusions

We developed stable and biocompatible photoluminescent BSA-Au-OCS nanocomplexes which fluoresce in the near infrared region. Positively charged OCS interacted electrostatically with BSA-AuNCs and enhanced the stability and safety. BSA-Au-OCS nanocomplexes exhibited strong fluorescence emission at ~ 620 nm. In addition, BSA-Au-OCS nanocomplexes were readily taken up by cells, evidenced by fluorescent imaging. BSA-Au-OCS nanocomplexes also exhibited strong fluorescence emission in tissues. The results suggest that BSA-Au-OCS nanocomplexes hold potential as a bioimaging agent.

## Abbreviations

Au NCs: Gold nanoclusters
BSA: Bovine serum albumin
BSA-Au-OCS: Oligochitosan-stabilized BSA-gold nanoclusters
MTT: 3-(4,5-Dimethylthiazol-2-yl)-2,5-Diphenyltetrazolium Bromide
OCS: Oligochitosan
TEM: Transmission electron microscopy
